# Disruption to the doctor–patient relationship in primary care: a qualitative study

**DOI:** 10.3399/BJGPO.2022.0039

**Published:** 2022-09-21

**Authors:** Kyle Eggleton, Nam Bui, Felicity Goodyear-Smith

**Affiliations:** 1 Department of General Practice & Primary Health Care, The University of Auckland, Auckland, New Zealand

**Keywords:** primary health care, general practice, COVID-19, professional–patient relations, patient care: delivery of health care

## Abstract

**Background:**

Starfield described the importance of system-level components of primary care (first contact, continuous, comprehensive, coordinated), on countries’ health systems. It is postulated that, at the individual level, interpersonal interactions and relationship-centred care are central to primary care.

**Aim:**

To explore the impact of COVID-19 on disruption to the doctor–patient relationship and subsequent development of new models of care.

**Design & setting:**

A series of 11 cross-sectional surveys of New Zealand (NZ) urban and rural primary care doctors, nurses, and managers, from May 2020 to February 2021, to understand and monitor responses to the pandemic.

**Method:**

Using inductive content analysis, cumulated qualitative data from doctors were examined through the lenses of the doctor–patient relationship, its disruption, and resulting changes in models of care.

**Results:**

There were 1519 responses to the surveys, representing 482 unique participants. The majority (86%) of responses were from doctors. The following four key themes emerged: moving to transactional consultations; task-shifting with team changes; creating a production line; and diminished communication and coordination across services.

**Conclusion:**

The advent of the pandemic led to severe and ongoing strain on practices requiring rapid change to the model of care. Team members took on new roles for triaging, testing, and separating patients with respiratory and non-respiratory symptoms. There was a rapid move to telehealth, with policies developed on where face-to-face consultations were necessary. Practice strain was exacerbated by disruption to coordination with secondary and other referral services. As new models of general practice develop, further disruptions to development of doctor–patient relationships must be avoided. This work extends Starfield’s system-level paradigm to the individual level, with the core value of primary care the doctor–patient relationship. Successful sustainable models are likely to be where relationships are treated as of central importance.

## How this fits in

First contact, continuity, comprehensiveness, and coordination are system-level components of primary care. The COVID-19 pandemic caused strain to primary care practices. At the individual level, the core value of primary care is the doctor–patient relationship. Disruption to this relationship during the pandemic stressed GPs but also led to new models of care.

## Introduction

Barbara Starfield’s groundbreaking work demonstrated the positive effect of primary care,^1^ which it described as a level of care within the country’s health system that provides ‘*first-contact, continuous, comprehensive, and coordinated care to populations undifferentiated by gender, disease, or organ system*’.^2^ This system-level approach is seen to be a critical component for countries to achieve universal health coverage.^3^ Starfield subsequently added the attributes of family- or patient-centredness, cultural competency, and community orientation to the core principles of first contact, continuity, comprehensiveness, and coordination.^4^

Starfield often used the terms ‘primary health care’ and ‘primary care’ interchangeably,^1^ but these concepts have since been refined and differentiated. The World Health Organization has defined primary health care as a system-level approach to service delivery, combining individual-based care and population-level public health, and including health promotion, disease prevention, public health, and rehabilitation services, as well as multi-sectoral functions including community-based social services.^5^ ‘Primary care’, involving first contact with patients and their families, is thus a subset of primary health care. While Starfield addressed first contact and patient-centredness as important attributes, her focus was predominantly at a population and system level. At an individual level, the present authors postulate that interpersonal interactions and relationship-centred care are central to primary care.

The COVID-19 pandemic that swept the world in early 2020 caused disturbance on many levels. Countries closed their borders and instigated a raft of public health measures — from mask-wearing, physical distancing, isolation and quarantine, and restriction of mass gatherings through to total lockdown mandates — to prevent or mitigate the spread of the SARS-CoV-2 virus. COVID-19 testing and contact-tracing systems were set up. In NZ, the first COVID-19 case was detected on 28 February 2020, the border closed on 20 March, and by 25 March the country had plunged into hard lockdown. Frontline primary care practices were hard hit on many levels, having to minimise in-person consultations, and triage and separate patients with respiratory symptoms into dedicated ‘red streams’ with staff wearing personal protective equipment (PPE). They struggled to access adequate COVID-19 tests and PPE, and government money was slow to come. Practices rapidly switched to telehealth consultations. Consultation numbers plummeted. It was reported that GPs were losing their jobs or working for free, and some practices closed.^6^

**Figure 1.**
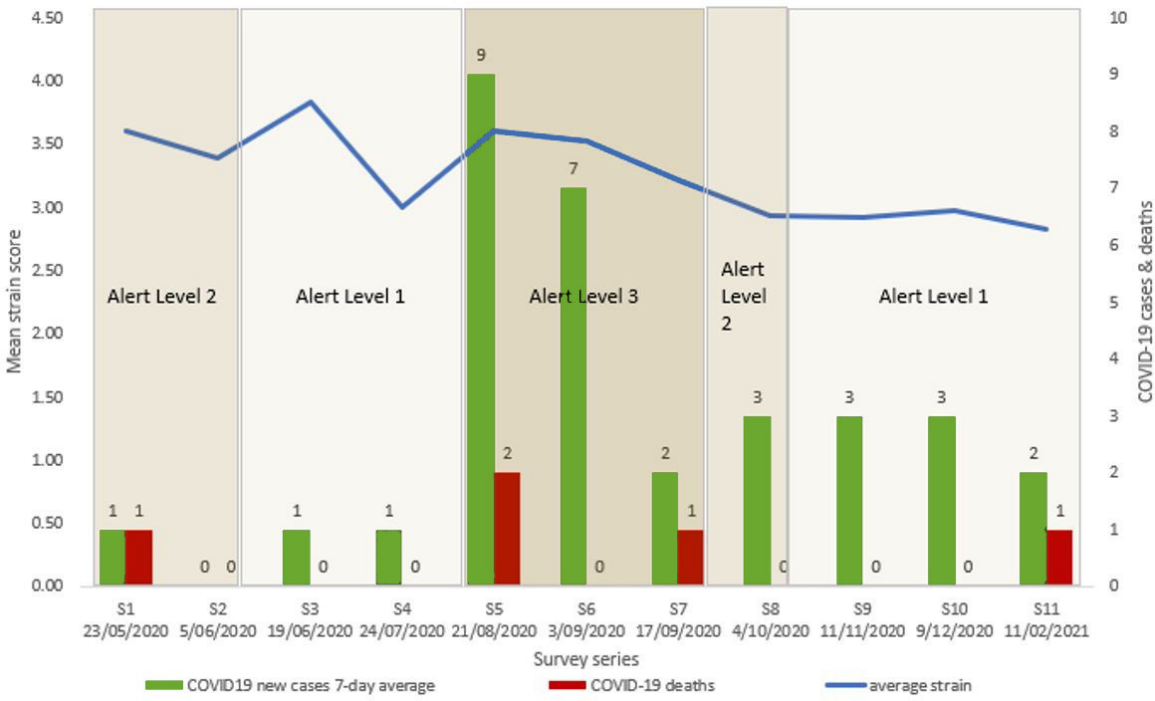
Mean strain scores, COVID-19 cases and deaths, and alert levels during survey series. New cases include both border arrivals in managed isolation and community spread. Alert levels are for Auckland: occasionally there was a lower level for the rest of the country.

In the 11-survey series (May 2020 to February 2021), previously reported^7,8^ and further described below, NZ general practices reported being under substantial strain, with practitioners suffering from considerable distress. The level of strain remained high across the series, ranging from 3.84/5 to 2.83/5, despite the fact that NZ eliminated community COVID-19 infections; had small case numbers; had only occasional, contained outbreaks; and had few deaths (Figure 1). Alert levels 3 and 4 were forms of lockdown, and under level 2 there were limits on gatherings. For long periods of time, the country was under alert level 1, with minimal restriction. Few practices encountered any COVID-19 cases during this period.

Certainly clinicians have extrinsic motivations, such as money and ‘doing it right’ for authority, but more importantly, they have intrinsic motivations around values and professionalism. It is hypothesised that their ongoing stress related more to the disruption of the clinical relationship than to external factors such as funding and resource constraints.

Preliminary studies have indicated that GPs found that the rapid move to telehealth, while essential to maintain some clinical connection, posed considerable issues in being able to communicate with many of their patients. In Italy, which was badly affected by the first wave of the pandemic, telehealth was seen by GPs as a way to stay emotionally connected with their patients, as long as they had an existing relationship.^9^ In another qualitative study, Danish GPs deemed telehealth acceptable to use short term in the COVID-19 crisis situation, but its use largely diminished as soon as it was viable to resume face-to-face consultations.^10^ Similarly, primary care doctors interviewed in Oman indicated that telehealth often resulted in suboptimal doctor–patient interactions.^11^ GPs in the US had concerns about inadequate patient care, lack of physical patient interaction, and technology isssues.^12^

On the other hand, in some studies patients viewed telehealth favourably. In Poland, patients surveyed generally indicated a high degree of satisfaction with their telehealth GP consultations.^13^ Interviews with Australian patients in general practice indicated that telehealth was more acceptable where there was an existing doctor—patient relationship, and should be continued but combined with opportunities for face-to-face consultations after the COVID-19 pandemic is over.^14^

This study aimed to explore the impact of COVID-19 on disruption to the doctor–patient relationship from the NZ GP perspective, and subsequent development of new models of care.

## Method

### Setting

In May 2020 a series of cross-sectional ‘Quick’ surveys were instigated of NZ primary care practices, which aimed to understand and monitor responses to, and difficulties experienced from, the pandemic. Similar surveys were independently run in Australia, the US, and Canada. Eleven surveys were conducted between May 2020 and February 2021. Surveys were the fastest means to collect data from practices across NZ about experiences, and rapidly disseminate the findings.

Participants were urban and rural primary care clinicians (doctors and nurses) and practice managers throughout NZ. Survey links were disseminated by NZ primary care organisations, including the Royal New Zealand College of General Practitioners, the Royal New Zealand College of Urgent Care, General Practice New Zealand, the New Zealand Rural General Practice Network, the Practice Managers and Administrators Association of New Zealand, as well as several other groups, such as primary care organisations and private medical Facebook group members, with invitations to snowball the request. It was not possible to target receptionists as they have no specific membership organisation, although in small practices the manager often also serves in this role.

Surveys contained five core questions, which included enquiring about the strain and types of stressors experienced, and use of face-to-face and telehealth consultations, with additional ‘flash’ questions exploring the most pressing information needs expressed by the sector. Findings were analysed immediately after the surveys closed. Summary findings and infographics were produced (https://covid-19-pc.auckland.ac.nz/results/) and disseminated back to the participating organisations, the Chief Science Adviser for the Ministry of Health, the Director-General of Health, and the NZ media. The project provided an opportunity for primary care practitioners to have their voices heard by policymakers. The methods have been published in full previously.^7,8^

### Analyses

The cumulated qualitative data from all 11 surveys were examined through the lenses of the doctor–patient relationship, its disruption, and resulting changes in models of care. Using an inductive content approach, the text was categorised and organised into themes through an abstraction process. For this analysis, the data were restricted to medical staff. The full dataset was coded by one researcher (NB) in NVivo (version 12) software, with a random selection independently cross-checked for coding strategy and data interpretation by a second (FG).

## Results

There were 1519 responses to the 11 surveys, representing 482 unique participants (many responders participated in multiple surveys). The vast majority (86%) were doctors, with 3% nurses, and 11% practice managers.

Four key themes emerged specifically relating to disruption to the doctor–patient relationship, which were as follows: moving to transactional consultations; task-shifting with team changes; creating a production line; and diminished communication and coordination across services.

### Moving to transactional consultations

The theme of ‘moving to transactional consultations’ related to how movement away from face-to-face consultations to remote consultations meant that consultations became more transactional and less performative. General practices shifted rapidly to a system of phone triage and telehealth consultations. While this was initially stressful, many participants discussed how the changes were positive and overdue. It appeared that many practices were contemplating changes to their model before the pandemic. Positive aspects of remote consultations were that there was more resilience, that telehealth enabled practices to be proactive and support their patients, and that triaging enabled some problems, such as repeat prescriptions, to be dealt with quickly:

‘*While the first few weeks were a strain on our staff and services, we have made some excellent new changes — many long overdue and will assist a much more resilient well-managed system in our future.’* (S11/160)

The rapid move to e-prescribing and electronic communications was often valued, with GPs able to email scripts to pharmacy, complete virtual work medical certificates, and communicate more rapidly with specialist services. Some population groups benefited from remote consultations, such as patients just needing a routine follow-up, busy working mothers, or rural patients:

*‘My rural patients love not having to leave their farms to come in.’* (S8/37)

However, there were also challenges for some population groups and certain situations, such as poor mobile phone coverage in rural areas, technology difficulties for older patients, and patients with poor information technology literacy. Other challenges included patients with English as a second language or other communication barriers such as impaired hearing:

*‘Our patients have English as second language, lack of IT knowledge provides further barriers.’* (S1/63)

Financial issues were a concern for many and related to reluctance by patients to pay, because they deemed telephone consultations not to have the same value as face-to-face interactions, or thought that they should not be charged for short telephone consultations:

‘*Patients often view telephone consultations as a lesser consultation. This results in a reluctance to pay.’* (S1/21)

The most significant challenge with telehealth, however, was disruption to the performative aspects of the consultation. GPs talked about how telehealth consultations were one-dimensional and transactional, that sometimes an important piece of information would be missed because they were unable to read patients’ body language, or pick up on other non-verbal cues. Phone delay might impede the conversation with patients talking over the GP:

*‘Telehealth is awful, no non-verbal cues, patients talk over you.’* (S2/15)

GPs commented on the difficulty in establishing therapeutic relationships over the phone, which were especially pronounced for patients with complex problems, high needs, or mental health issues, and for new, child, or older patients. They identified many circumstances and conditions not suitable for telehealth consultations such as:

*‘Unstable diabetes. Poor health literacy. Acute asthma, uncontrolled hypertension, AF CHF* [atrial fibrillation, congestive heart failure]*, unstable mental health (suicidal intentions) multiple long-term conditions. Cognitive impairment concerns. New malignant diagnosis.’* (S9/36)

*‘Older, tech naive, deaf patients with more major ailments requiring physical exam, babies and toddlers.’* (S9/27)

*‘Seeing patients that you don't already know.’* (S9/3)

### Task-shifting with team changes

There was rapid task-shifting and changes within practice teams to support the development of new models of healthcare delivery. Changes involved upskilling receptionists and shifting tasks to nurses. One of the key changes was training receptionists how to conduct telephone triage to determine whether patients needed GP consultations, or could be directed elsewhere, such as to a nurse or an online portal. Receptionist triage led to more appropriate use of GPs’ time:

*‘Receptionists have all been trained in tele-triage resulting in better use of GPs’ consultation timeslots during the day.’* (S2/114)

One of the outcomes of task-shifting was an increase in nursing workload. Nurses were undertaking more consultations and were also heavily involved with COVID-19 responses, which took them away from regular nursing duties:

*‘Nursing staff tied up with COVID-19 testing so less available for general work.’* (S5/116)

The changes, from a GP perspective, were generally viewed positively and strengthened the team dynamic, bringing team members closer together.

### Creating a production line

Part of the task-shifting involved running separate respiratory clinics. Mechanisms were put into place to triage, separate patients, and increase efficiencies in the face of a significant workload. Practices had to rapidly triage patients into ‘green’ and ‘red’ streams (patients with respiratory or possible COVID-19 symptoms) to prevent infection spread. Patients in the red stream had to be seen separately with clinicians wearing full PPE. Keeping patients with respiratory symptoms separate added extra pressure on reception and nursing staff, as well as GPs:

‘*We had to change the way we do things. Keeping respiratory patient separate from others. More pressure on reception nursing staff as all patients need triage. Had to hire a cabin to see respiratory patients in.’* (S4/78)

Various aspects to this change included the need for practices to quickly create their own solutions for managing the different streams such as purchasing or renting cabins. New processes, systems, and solutions were created, and this required a degree of agility for practices. It appeared that the new systems improved efficiencies and resulted in increased outputs:

*‘Our drive-through flu vaccination clinics were amazing. We reduced appointment times from 10 min to 5 min to 2 minutes! It was so much easier and safer for elderly patients especially.’* (S11/96)

### Diminished communication and coordination across services

The final theme of ‘diminished communication and coordination across services’ captured the sentiment by participants that even though practices experienced increased communication and collaboration within their teams, there was a distressing lack of support from, and coordination with, other services. Services that were delivered by District Health Boards (DHBs) were notable for their lack of general practice support. These services included district nurses, community mental health services, and secondary care specialists. Participants, for example, commented that hospital referrals were being declined more than usual. However, not only DHB services were lacking, but also social support providers:

*'Community mental health and social support providers not able to provide their previous level (just-enough) services … mortifying and heartbreaking for us GPs to be talking up a previously reliable service to patients and on referring them only to find they are not picked up/no communication and we have an even more distressed mess to wade through two months down the line.*’ (S6/153)

A consequence of the lack of support was that practices had to deal with increasing workload to manage patients, and be proactive.

## Discussion

### Summary

The COVID-19 pandemic led to severe and ongoing strain on practices, necessitating rapid change to the model of care and a paradigm shift in the way clinicians practised. Practice strain was exacerbated by disruption to their coordination with other supporting and referral services, including secondary care. GPs were stressed by the disruption to the normal way they were able to relate to their patients, and their inability to ensure that all non-COVID-19 conditions were addressed both by themselves and by other providers to whom they referred patients.

However, over time new models of care and ways to maintain the doctor–patient relationship developed. Team members took on new roles for triaging, testing, and separating patients with respiratory and non-respiratory symptoms. There was a rapid move to telehealth, with policies developed for those who needed to be seen face to face.

### Strengths and limitations

The Quick COVID-19 surveys were designed to be a pragmatic and rapid means to inform public policy on the impact of COVID-19 on primary care, and hence were not necessarily representative of all practices. Although receptionists are members of the primary care team, they could not be targeted as participants. However, a rich qualitative dataset was generated from 11 surveys spanning 10 months, and robust thematic analyses were conducted, applying the principles of primary care theoretical framework.

### Comparison with existing literature

Substantial disruption to routine primary care provision occurred globally during the pandemic.^15^ The rapid uptake of digital technology, including virtual consultations, e-prescriptions and e-referrals, became a critical component of providing ongoing care.^16^ For simple transactions, both GPs and patients have come to recognise the value and convenience of telehealth.^17^ International evidence confirms that older patients, those with visual, hearing, or cognitive impairment, those from ethnic minority and rural backgrounds, and the socially deprived are less likely to be reached by telehealth services, risking increasing health disparities.^18–21^ However, in NZ a study showed that telehealth actually reduced access barriers for indigenous Māori.^22^

Although Starfield’s definition of primary care related to system level attributes, there was evidence that the pandemic-induced changes in the general practice model reflected attempts to maintain some of these attributes at a practice level. Practices coordinated health care despite lack of support from other services. Comprehensiveness was seen in the additional services provided, such as rapid respiratory and vaccination clinics. First point of contact was maintained through task-shifting and triage and facilitating remote consultations. However, the changes to the model, although increasing efficiencies, also created tensions in terms of developing and suataining relationships with patients. While the Starfield model does not specify relationships as an important attribute of primary care, the disruption to practice, as demonstrated by this study, reveals this to be a critical characteristic.

Shifting from face-to-face consultation to telehealth requires significant adaptation in how doctor–patient relationships are generally conducted. The authors believe that the disruption of the doctor–patient relationship through virtual consulting was exacerbated by lack of all the non-verbal cues and performative components of the consultation.

Assessing a patient begins by seeing how they walk from the waiting room. The physical examination may be an important means of communication beyond its diagnostic value. A patient’s blood pressure may be taken partly because it allows the clinician to provide reassuring physical contact. The intimacy of contact from a touch on the hand or shoulder may be as important to patients who live alone as the medical aspects of the consultation. The practice of medicine is both a science and an art.^23^ De Zulueta stated *‘touch can help us as clinicians to discern, detect, and diagnose, but can also allow us to express empathy, reassurance, comfort, and presence.’* ^24^ Touch has the power to soothe and to heal.^25,26^ Peloquin described it as the embodiment of caring.^27^ The concept of the healing touch, the *‘laying on of hands’* goes back to antiquity.^28^

A study of patients found that they considered expressive touch on the hand or forearm as acceptable, especially when they were distressed, and most GPs agreed, although some worried that this might be misinterpreted.^29^ In another study, patients reported that they found touch on the hand or shoulder comforting.^30^ The disruption of the doctor–patient relationship caused by telehealth was a major source of distress experienced by the GPs, but they also recognised that not having to travel to the practice suited some patients, and developed new models of care accordingly.

From the findings, the authors postulate an extension of Starfield’s work which looks at system-level primary care factors such as continuity and comprehensiveness,^31,32^ to the individual level where the core value is the doctor–patient relationship. Primary care connects the scientific and the social. It requires connection and communication with patients, collective decision-making, and cooperative care. The pandemic disrupted this relationship in many ways, requiring significant adaptation to how these relationships are generally conducted. General practice is resilient, and as solutions were found to address stressors caused by COVID-19, new models of care evolved.

### Implications for research and practice

As new models of general practice develop, it is important to avoid further disruptions to the development of relationships between patients and doctors. Successful and sustainable models are likely to be those in which relationships are treated as of central importance. For consultations requiring complex diagnoses, problem-solving, or instigating new interventions that are likely to be long term, meeting patients face-to-face can provide valuable non-verbal cues, the ability to use diagnostic and therapeutic procedures, and opportunities offer reassurance and comfort through physical touch. However, for more routine issues, both providers and patients value the convenience of virtual consultations, and this mode of care will remain useful beyond the pandemic.^17,33^

Further research is needed in this rapidly changing area of primary care delivery to understand the challenges and the benefits of telehealth to both providers and patients. The authors propose series of case studies to determine which models of care developed by Aotearoa New Zealand primary care practices are best adapted to deal with the COVID-19 pandemic, including practices catering for vulnerable populations such as Māori, Pacific, rural, and those of low socioeconomic status. This will inform support and investment in new models of care to improve equitable access to primary care services and reduce the impact of future pandemics.

## Data

It is not suitable for the data to be made publicly available.
